# Physics-Informed Neural Network with Residual Correction Architecture for Hybrid Feedforward–Feedback Temperature Control of DFB Semiconductor Lasers

**DOI:** 10.3390/s26123869

**Published:** 2026-06-18

**Authors:** Xiongfei Yin, Sicheng Sun

**Affiliations:** 1 School of Physics and Photoelectric Engineering, Key Laboratory of Gravitational Wave Precision Measurement of Zhejiang Province, Taiji Laboratory for Gravitational Wave Universe, Hangzhou Institute for Advanced Study, University of Chinese Academy of Sciences, Hangzhou 310024, China; sunsicheng23@mails.ucas.ac.cn; 2 University of Chinese Academy of Sciences, Beijing 100049, China

**Keywords:** physics-informed neural network, DFB laser, temperature control, thermoelectric cooler, residual correction, hybrid control, data efficiency

## Abstract

Wavelength stability of distributed feedback (DFB) semiconductor lasers in dense wavelength division multiplexing (DWDM) systems hinges on sub-millikelvin temperature regulation, a task complicated by the nonlinear, multi-node dynamics of the thermoelectric cooler (TEC) and the purely reactive nature of conventional proportional–integral–derivative (PID) control. We present a physics-informed neural network (PINN) built around a residual correction architecture for hybrid feedforward–feedback TEC temperature control. Rather than penalizing physics-residual violations in the loss function, the architecture wires a simplified one-node thermal model directly into the network graph as a frozen baseline. A trainable branch then learns only the residual mismatch. Temporal lag features are appended to the input so that the network can reconstruct unmeasured internal thermal states from the cold-side temperature history, which proves essential for overcoming the partial-observability bottleneck inherent in multi-node TEC packages. Ablation experiments on a high-fidelity three-node TEC simulator show that all model variants (PINN, physics-feature-augmented NN, and pure NN) exceed R^2^ = 0.993 when trained on the full dataset, yet the PINN’s advantage becomes pronounced under data scarcity. At a 3% training budget, it reaches R^2^ = 0.966 versus 0.930 for the pure NN, implying an approximately 5.4× reduction in the data needed to reach a given accuracy target. In closed-loop validation, the PINN+PID hybrid settles 60% faster than standalone PID. Tracking RMSE drops by 69%, and peak disturbance deviation falls by 74%, across step, multi-setpoint, and current-perturbation scenarios. All results reported here are obtained in simulations. Experimental validation on physical DFB-TEC hardware is left to future work.

## 1. Introduction

Distributed feedback (DFB) semiconductor lasers serve as the workhorse transmitter in dense wavelength division multiplexing (DWDM) networks owing to their single-longitudinal-mode operation and narrow spectral linewidth [1,2]. A well-known vulnerability is that the lasing wavelength shifts by approximately 0.1 nm/K for typical InGaAsP chips around 1550 nm [3]. A 100 GHz ITU channel grid leaves almost no margin. The thermal excursion of ±0.01 K is enough to push the carrier outside its slot [4]. Wavelength stabilization within this window is the task of the thermoelectric cooler (TEC) inside the butterfly package. Holding sub-10 mK stability under real operating conditions is harder than first-order thermal analysis suggests.

The TEC current and the junction temperature are coupled nonlinearly through the Peltier effect. The *I^2^R* Joule term partially cancels the Peltier cooling. The optimum drive point, therefore, shifts with the operating condition [5]. The heat-flow path inside a butterfly package crosses several thermal masses. These include the submount carrying the laser chip, the ceramic spacer beneath it, and the case-mounted heat sink on the hot side. Their time constants span roughly 0.1 s to a few seconds. A single-node lumped model cannot reproduce even the step response [6]. Conditions on a deployed line card are worse. Ambient drift, thermal crosstalk from neighboring lanes, and step changes in laser bias current all act as disturbances. The TEC loop must reject them at the same time.

In practice, almost every commercial laser driver still ships with a plain PID loop [7,8]. Steady-state performance is adequate, but PID is inherently reactive. The integrator only starts accumulating after an error appears. Step the setpoint by 2 K or bump the laser bias current, and the controller is already behind. The result is the familiar overshoot-then-ring pattern. Adding a feedforward branch changes the picture fundamentally, because a model that estimates the required TEC current ahead of time offloads the bulk of the transient work, leaving the PID to mop up whatever residual the model misses [9,10].

We adopt a residual correction architecture in which the simplified physics model enters as a structural component of the network rather than as a regularization term in the loss. The network output is formed as the sum of a frozen single-node prediction and a trainable correction, with the correction weights initialized near zero. When training data are scarce, the correction remains small, and the model reduces to the analytical baseline. As more labeled samples are accumulated, the correction branch progressively accounts for the nonlinearities that the single-node formula cannot represent. Temporal context is incorporated in parallel. Two past samples of cold-side temperature and TEC current, taken 0.5 s apart, are appended to the input vector. These lagged values provide indirect access to the ceramic-layer and hot-side temperatures, neither of which is instrumented. Removing them caused every architecture we evaluated to saturate at R^2^ ≈ 0.77.

The contributions of this work are as follows. First, we propose a residual-correction PINN in which the one-node thermal model is embedded directly into the computation graph with frozen weights, avoiding the gradient conflict between physics and data losses that limits the accuracy of conventional PINNs when the model mismatch is large. Second, we find that appending only two lagged temperature–current pairs to the input is sufficient to overcome the partial observability of single-sensor TEC packages. Without these features, none of the architectures we evaluated exceeded R^2^ ≈ 0.77. Third, a data-budget sweep from 3% to 100% of the available training set shows that the PINN reaches a given accuracy target with about 5.4× fewer labeled samples than an equivalent purely data-driven network (R^2^ = 0.966 at 293 samples, compared with R^2^ = 0.930 for the NN). Finally, the trained surrogate is embedded in a hybrid feedforward–PID controller and benchmarked in closed-loop simulation against a standalone PID, an integral-separated PID, and an NN+PID baseline across step, multi-setpoint, and disturbance-rejection scenarios, with the plant represented by a three-node TEC model.

Section 2 below surveys related work on TEC control, PINNs, and hybrid physics–data methods. Section 3 lays out the thermal models and the proposed architecture. Section 4 details the experimental protocol, Section 5 reports the results, and Section 6 concludes.

## 2. Related Work

### 2.1. TEC Temperature Control

The earliest TEC controllers were analog PID loops. Digital implementations later offered greater flexibility in tuning [7], and a number of variants have since been reported. Zhang et al. [11] applied a variable-domain fuzzy PID scheme to a semiconductor laser. The method achieved regulation over a wide range, from −12 °C to 120 °C. Steady-state accuracy, however, remained modest. Liu et al. [12] combined mechanism-based system identification with a fuzzy PID controller for semiconductor laser modules. They reported a temperature stability of 0.02 °C over four hours. Real-time optimization-based controllers have also been studied [13]. In practice, they require dedicated FPGA hardware to meet the sampling-rate demands of a laser-module thermal loop.

Analytical TEC models fail to capture much of the system nonlinearity. Attention has therefore shifted toward methods that rely less on first-principles description. Wang and Xi [14] designed an integrated current-drive and temperature-control circuit with refined PID tuning. The method is effective in operation. It remains model-free, however, and does not exploit the known physics of the TEC. Purely empirical models share a common limitation. They require large, labeled datasets for training. They also tend to generalize poorly once the operating point moves outside the training range.

### 2.2. Physics-Informed Neural Networks

The PINN framework was introduced by Raissi et al. [15], who added a PDE-residual term to the data-fitting loss so that the trained network is constrained to satisfy the governing equations in addition to the measurements. The approach was taken up in a range of disciplines, including computational fluid dynamics [16], heat transfer [17], and solid mechanics [18]. Cai et al. [19] showed that sparse thermocouple measurements are sufficient for full-field temperature reconstruction when the conduction equation is enforced as a constraint, and later studies have extended PINNs to transient problems and to coupled multi-physics settings [20]. Comprehensive surveys of physics-informed machine learning and its applications provide broader context for these developments [21,22,23].

Model mismatch remains a central difficulty for PINNs in real hardware settings. In packaged thermal devices, the encoded equations are necessarily approximate, and when the mismatch is large, the gradient from the physics term drives the optimizer away from the data-optimal solution [24]. Several mitigation strategies have been reported in the literature. One option is to treat the equation coefficients as trainable parameters [25]. Another is to decompose the domain into subregions, as in XPINNs [26]. A third is to learn an additive correction on top of an analytical baseline [27]. The method developed in this work follows the residual formulation of [27], with two modifications tailored to TEC thermal control. The analytical baseline is specialized to a single-node cooler model, and lagged measurements of cold-side temperature and TEC current are added to the input to compensate for the unobserved internal states of multi-node packages.

### 2.3. Hybrid Physics–Data Control

Hybrid physics–data controllers have appeared in several adjacent thermal domains. Gokhale et al. [28] injected physics-based structural priors into a recurrent neural network aimed at building-level temperature prediction and showed measurable generalization gains when labeled data were scarce. Drgona et al. [29] enforced thermodynamic constraints inside a deep learning controller for multi-zone HVAC. Most relevant to us, Kim et al. [30] combined a Peltier equation with an NN to predict TEC temperature in a laser module. Where our work departs from these studies is in two specifics. First, we keep the physics model and the learned correction structurally separate at the output, rather than mixing them in the loss. Second, we report not just peak accuracy but a controlled data-efficiency sweep, which none of the above papers provide. We are also not aware of prior work that uses temporal lag features to infer hidden node temperatures inside a TEC package.

## 3. Methodology

### 3.1. TEC Thermal Models

#### 3.1.1. Simplified One-Node Model

We start from a single-node energy balance for the cold side (temperature T):

(1) $$C\;\cdot \;\frac{dT}{dt}\;=\;-\alpha IT\;+\;K\left(T_{a}\;-\;T\right)\;+\;\frac{1}{2}I^{2}R\;+\;Q_{laser}\;-\;hA\left(T\;-\;T_{a}\right)$$

Here, C is the lumped thermal capacitance, α the Seebeck coefficient, I = −u the TEC drive current, K the thermal conductance between the cold side and ambient, R the electrical resistance, *T*_a_ is the ambient temperature, *Q* is the laser heat load, h the convective coefficient, and A the exposed surface area. This formula captures the dominant energy flows but says nothing about the ceramic interlayer or the hot-side heat sink. How much that omission matters will become clear when we introduce the three-node plant.

#### 3.1.2. High-Fidelity Three-Node Model

A three-node formulation is used to capture the dynamics not resolved by the one-node model, with separate thermal masses for the cold side ($T_{c}$), the ceramic/intermediate layer ($T_{m}$), and the hot side ($T_{h}$). The material properties of the thermoelectric elements (the Seebeck coefficient, thermal conductance, and electrical resistance) depend on the local temperature. They are represented by quadratic polynomial fits. The temperatures of the three nodes are governed by the following coupled ODEs:

(2) $$C_{c}\;\cdot \;\frac{{dT}_{c}}{dt}\;=\;-\alpha \left(T_{c}\right)\cdot I\cdot T_{c}\;+\;K_{1}\left(T_{m}\;-\;T_{c}\right)\;+\;\frac{1}{2}I^{2}R\left(T_{c}\right)\;+\;Q_{laser}\;+\;\varepsilon$$

(3) $$C_{m}\;\cdot \;\frac{{dT}_{m}}{dt}=K_{1}\left(T_{c}-T_{m}\right)+K_{2}\left(T_{h}-T_{m}\right)+K_{ct}\cdot \left(T_{h}-T_{c}\right)$$

(4) $$C_{h}\;\cdot \;\frac{{dT}_{h}}{dt}=\alpha \left(T_{h}\right)\cdot I\cdot T_{h}+K_{2}\left(T_{m}-T_{h}\right)+\frac{1}{2}I^{2}R\left(T_{h}\right)-hA\left(T_{h}-T_{a}\right)$$

We use this three-node model as our virtual test bench throughout. It provides all training labels and serves as the plant for closed-loop evaluation. A key constraint is that the controller sees only $T_{c}$. The intermediate and hot-side temperatures $T_{m}$ and $T_{h}$ are never measured, which poses a genuine observability challenge. Two physically distinct internal thermal configurations can map to the same observed cold-side reading yet lead to quite different values of $\frac{{dT}_{c}}{dt}$ for the same observed ($T_{c}$, u).

### 3.2. PINN with Residual Correction Architecture

#### 3.2.1. Architecture Design

**Figure 1:**
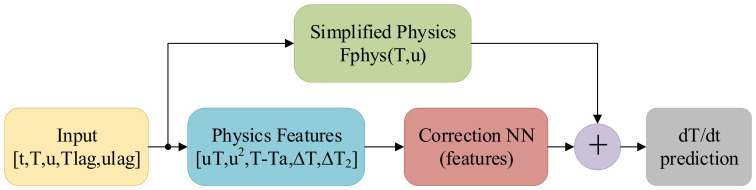
Architecture of the proposed residual-correction PINN. The frozen one-node physics model supplies a baseline, and the trainable correction branch captures the remaining mismatch. Selectively switching the physics base and engineered features on or off yields the three model variants compared in this study (PINN, PhysFeat, and NN). Here, “+” denotes the element-wise summation of the frozen physics baseline and the learned correction, as defined in Equation (5).

We split the predicted derivative into two additive terms, the physics baseline and a learned correction (Figure 1):

(5) $$\frac{dT}{dt}\;=\;f_{physics}\left(T,\;u\right)\;+\;g_{\theta }\left(x\right)$$

where $f_{physics}$ denotes the one-node model of Equation (1) with fixed parameters, and *g_θ_* is a neural network with trainable parameters *θ*. This formulation differs from a standard PINN in one key respect. In the conventional approach, the physics enters as an additional term in the loss. Here, it is embedded directly in the computation graph. When the analytical model deviates from the true system, the physics-residual term produces gradients toward a solution consistent with the incorrect equations. These gradients oppose those from the data term. Freezing the physics branch removes this conflict. The optimizer sees only the data loss and a light regularizer on the correction magnitude. Because the governing relation is embedded structurally in the network graph rather than imposed as a soft penalty in the loss, the present architecture is more precisely described as physics-embedded than as a conventional physics-penalized PINN. We retain the term PINN for continuity with the literature while making this distinction explicit.

There is a useful bonus. The correction $g_{\theta }$ is typically smaller in magnitude and smoother than the full $\frac{dT}{dt}$ signal, so the function the network must approximate is simpler. To reinforce this, the output layer of *g* starts near zero, meaning an untrained model simply outputs $f_{physic}$. A vanilla NN has no such fallback and can produce nonsensical predictions when data are limited.

#### 3.2.2. Input Features with Temporal Context

The raw input vector is [t, T(t), u(t), T(t−Δ), u(t−Δ), T(t−2Δ), u(t−2Δ)], where Δ = 0.5 s is the lag interval. These past values are what allow the network to work around the hidden-state problem described in Section 3.1.2. The finite differences T(t) − T(t−Δ)  and T(t) − T(t−2Δ) act as proxies for the recent thermal slope and curvature, implicitly encoding information about $T_{m}$ and $T_{h}$. Strip away these lagged readings and identical (T, u) pairs can legitimately map to multiple $\frac{dT}{dt}$ targets, a source of variance that no amount of model tuning can eliminate.

The PINN variant receives five extra hand-crafted channels on top of the raw inputs: u·T capturing the Peltier coupling, $u^{2}$ for the Joule term, T − $T_{a}$ representing the thermal gradient, and two temperature-difference features, bringing the total to 12 input dimensions. In contrast, the pure NN sees only the 7 raw channels.

#### 3.2.3. Network Architecture

The correction network $g_{\theta }$ is a four-layer fully connected network whose hidden widths are 128, 128, 128, and 64, all with tanh nonlinearities throughout. Where two consecutive layers have equal width, a residual skip connection feeds the layer input directly to its output. The total parameter count is approximately 76,000. We initialize hidden-layer weights with the Xavier scheme. For the output layer only, weights are sampled from a narrow Gaussian (σ = 0.01) so that the correction starts near zero, consistent with the design rationale outlined above.

#### 3.2.4. Training

The training objective minimizes

(6) $$L=L_{data}+\lambda_{corr}\;\cdot \;L_{correction}$$

where $L_{data}$ is the MSE between predicted and target corrections, and $L_{correction}$ is a regularization term encouraging small corrections, with $\lambda_{corr}$ = 0.01. We train with Adam (lr = 10^−3^), halving the learning rate via ReduceLROnPlateau whenever validation loss stalls for 30 epochs. Weight decay is set to 10^−5^, and training halts automatically if the validation metric fails to improve for 100 consecutive epochs. Every input feature is z-score-normalized prior to training. Optimization uses mini-batches of size 256, and training is capped at 1000 epochs, with the early-stopping criterion above typically halting well before this cap. Together with the layer widths, activations, parameter count, optimizer, and learning-rate schedule specified in Section 3.2.3 and above, these settings fully determine the model and permit independent reproduction.

### 3.3. Hybrid Feedforward–Feedback Controller

The hybrid controller computes the TEC drive signal as

(7) $$u\left(t\right)=\alpha_{ff}\;\cdot \;u_{ff}\left(t\right)+\alpha_{fb}\;\cdot \;u_{fb}\left(t\right)$$

where $u_{ff}$ is the PINN-based feedforward term, and $u_{fb}$ is the PID feedback output. Each control cycle, we invert Equation (5) numerically. We search for the current u that would produce the desired $\frac{dT}{dt}$ (dictated by the setpoint trajectory) via a bounded bisection over the allowable current range. Because the physics term is monotonic in u over the normal operating range, the bisection always converges. Typical runs need 10–15 iterations.

Instead of using a fixed feedforward/feedback split, the weights $\alpha_{ff}$ and $\alpha_{fb}$ change dynamically with the current tracking error. During large transients (|e| > 1.0 K), the PID path dominates ($\alpha_{fb}$ = 0.8) so that the PID drives hard toward the setpoint. Below 0.05 K, the feedforward branch dominates ($\alpha_{ff}$ = 0.8) because the surrogate is more precise for small corrections. A linear ramp interpolates between the two regimes. We also apply integral separation in the PID whenever the error is large, which prevents windup during the first few seconds of a transient.

## 4. Experimental Setup

### 4.1. Data Generation

We generated training data by running 80 separate simulations of the three-node plant. Initial cold-side temperatures were drawn from ($T_{c}\in \;\left[15,\;40\right]\;{}^{\circ}C$) and control signals (step, sinusoidal, ramp, telegraph, chirp, and random walk; $u\;\in \;\left[-1.5,\;1.5\right]\;A$). Individual runs last between 30 and 120 s at a base sampling rate of 0.1 s. We then decimate by a factor of five to weaken temporal correlation among neighboring samples. We obtain the $\frac{dT}{dt}$ targets by finite differencing. Gaussian measurement noise (σ=2 mK) is superimposed on $T_{c}$. After pooling and shuffling all runs, we have about 12,200 usable samples. And 20% of the dataset is for validation, and the other 80% for training.

### 4.2. Ablation Design

To isolate what each physics ingredient actually contributes, we train three model variants that share the same backbone architecture (Table 1). The full PINN keeps everything: frozen physics baseline, hand-crafted physics features, and the correction regularizer. PhysFeat removes the physics baseline but retains the engineered input features. This shows whether feature engineering alone can replace structural physics embedding. The pure NN uses no physics. It learns everything from the seven raw inputs. All three models share the same layer widths, activation, learning-rate schedule, and early-stopping rule. Any accuracy gap must, therefore, come from the physics components.

### 4.3. Control Experiment Configuration

Four controllers are tested in closed loop on the three-node plant at 100 Hz. They are a conventional PID, an integral-separated PID (IS-PID), an NN+PID, and the proposed PINN+PID. All PID instances use the same gains ($K_{p}=\;1.5,\;K_{i}=\;0.08,\;K_{d}=\;0.3$). Three test conditions are used. The difficulty increases across them. First, a single 2 K setpoint step from 25 °C to 27 °C. Second, a four-transition tracking sequence with both heating and cooling segments. Last, the disturbance-rejection test, and ±0.4 A current pulses are injected into the TEC at preset instants.

## 5. Results and Discussion

### 5.1. Surrogate Model Accuracy

**Figure 2:**
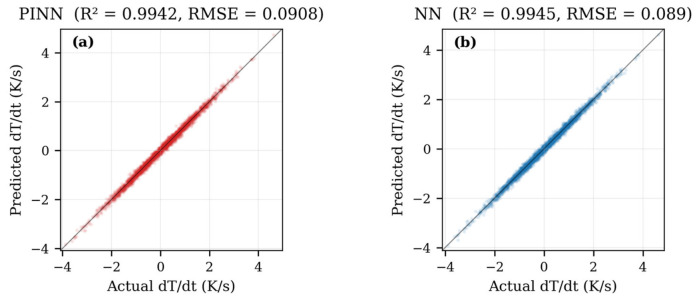
Validation-set scatter plots. (**a**) PINN. (**b**) Pure NN. Points cluster tightly around the identity line in both cases. It indicates the temporal features can resolve the hidden-state ambiguity for either architecture.

Table 2 lists the validation metrics. All three models were trained on the full dataset. Every variant achieves R^2^ = 0.993. The lag features alone resolve enough of the hidden-state ambiguity. And the physics-free NN can track the three-node dynamics accurately. Figure 2 shows the validation-set scatter plots for the PINN and the pure NN; in both cases the points cluster tightly around the identity line.

On the full dataset, the NN slightly beats the PINN. This might suggest that the physics term adds nothing. The reason is 9700 points already overdetermine the mapping. The frozen baseline adds an inductive bias. At this scale, it no longer helps. The physics branch only pays off when data are scarce. The next subsection shows this.

### 5.2. Data Efficiency Analysis

**Figure 3:**
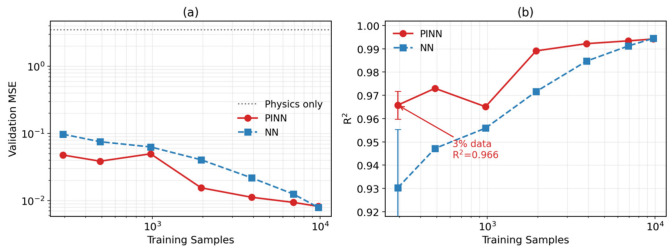
Data efficiency comparison. (**a**) Validation MSE versus training-set size on log–log axes. (**b**) Corresponding R^2^ curves. The PINN outperforms the NN at all small training budgets. The largest gap is 0.036 in R^2^, observed at the 3% budget. The two curves approach each other as the training set grows and become nearly indistinguishable at full size.

In the data savings task, the PINN and the NN were retrained on subsets ranging from 3% to 100% of the training pool. The validation set was held fixed. Table 3 and Figure 3 report the results.

The R^2^ curves separate as the training pool shrinks. At the 3% budget (293 samples), the PINN reaches R^2^ = 0.9658. The NN reaches only 0.9303. In MSE terms, the PINN’s MSE is about 51% lower than the NN’s. The cross-budget view is more direct. The PINN at 3% already beats the NN at 10%. The frozen energy-balance baseline, therefore, acts as a prior that is worth about 5.4× the labeled sample count. At 20%, the PINN reaches R^2^ = 0.9891, close to its full-data value. The NN at the same budget reaches only 0.9716.

The mechanism is straightforward. Because the frozen one-node branch already supplies a rough but physically grounded estimate, the correction network only needs to approximate a small, relatively smooth residual. The NN, in contrast, must reconstruct the full input–output relationship from raw features. Learning a smaller function takes fewer examples.

To assess the statistical reliability of the data-scarce regime, we repeated the 3% training-budget experiment over 10 independent random seeds and report the mean and standard deviation rather than a single run. The residual-correction PINN attains R^2^ = 0.966 ± 0.006, whereas the pure NN reaches only 0.930 ± 0.025. Two consequences follow. First, beyond its higher mean, the PINN exhibits roughly fourfold smaller seed-to-seed dispersion (standard deviation: 0.006 against 0.025). Because the test-set sampling error is an order of magnitude smaller still (±0.0005 and ±0.001, respectively), this spread originates almost entirely from training-sample selection and weight initialization rather than from the validation set. The lower variance is itself a useful result. Since the residual branch need only learn a small correction on top of the frozen physics baseline, its effective degrees of freedom, and, hence, its sensitivity to the random seed, are markedly lower than those of the unconstrained NN, which must reconstruct the full mapping. Second, we make the data-efficiency claim precise by defining the factor η(τ) as the ratio of the training-sample counts that the NN and the PINN respectively require to first reach a target accuracy R^2^ = τ. At the target τ = 0.9658, which is the accuracy the PINN already reaches at the 3% budget of 293 samples, the NN needs about 1592 samples, obtained by linear interpolation between the 10% and 20% budgets, so that η = 1592/293 ≈ 5.4×. One caveat applies to this number. The PINN target is a 10-seed mean, but the NN reference points at the 10% and 20% budgets are single runs, so η inherits their run-to-run variability and should be read as a point estimate rather than an exact multiplier. Given the seed dispersion of the NN at small budgets, we, therefore, describe the data-efficiency gain as roughly fivefold, with η ≈ 5.4× at this particular accuracy target, in place of the looser 5–7× figure used in earlier drafts.

### 5.3. Role of Temporal Features

**Figure 4:**
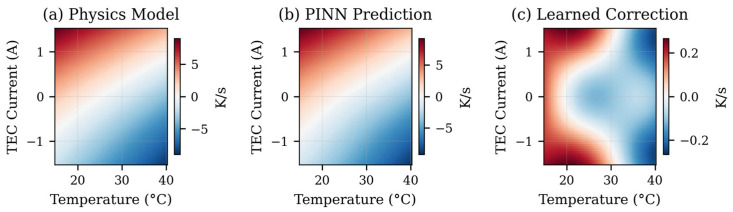
Learned correction. (**a**) One-node physics model. (**b**) Full PINN output. (**c**) Correction term only. The correction is largest at high currents. This is where Joule heating dominates, and the one-node model fits worst.

We retrained every model with the instantaneous triplet [t, T(t), u(t)] only, without any lagged samples. The result was unequivocal. PINN, PhysFeat, and NN all plateaued at R^2^ ≈ 0.77, and no amount of capacity increase or regularization tuning budged the number. This ceiling is not a training artifact. It reflects a hard information-theoretic limit. The controller has access to $T_{c}$, and the three-node system has two hidden states ($T_{m}$, $T_{h}$). Different internal configurations can yield identical (T, u) pairs yet produce different values of $\frac{{dT}_{c}}{dt}$. Without past readings to disambiguate, the mapping from (T, u) to the derivative is genuinely multi-valued, and no network, regardless of depth or width, can collapse that ambiguity. Figure 4 illustrates the learned correction: it is largest at high currents, where Joule heating dominates and the one-node model fits worst.

With the lagged snapshots added, every model rises above R^2^ = 0.99. The differences serve as numerical proxies for the first and second time derivatives. Together with the present state, they span the observable subspace of the three-node plant. One change in the input design lifts R^2^ from 0.77 to above 0.993. The binding constraint was observability, not model architecture.

Appending just two historical snapshots, separated by 0.5 s, instantly lifts every model above R^2^ = 0.99. The finite differences $\Delta T_{1}\;=\;T(t)\;-\;T(t-\Delta )$ and $\Delta T_{2}\;=\;T(t)\;-\;T(t-2\Delta )$ serve as numerical proxies for the first and second time derivatives, and together with the present-time state, they span the observable subspace of the three-node plant. The fact that a single change in input design, namely, adding two lagged columns, lifting R^2^ from 0.77 to above 0.993, underscores the point that observability, not architectural sophistication, was the binding constraint.

### 5.4. Control Performance

A high open-loop R^2^ does not by itself guarantee closed-loop performance. Errors in the feedforward prediction pass through the bisection inversion. They then couple with the PID dynamics. The resulting amplification depends on the scenario. Closed-loop testing is the only reliable check. Table 4 summarizes the four controllers across the three test conditions.

**Figure 5:**
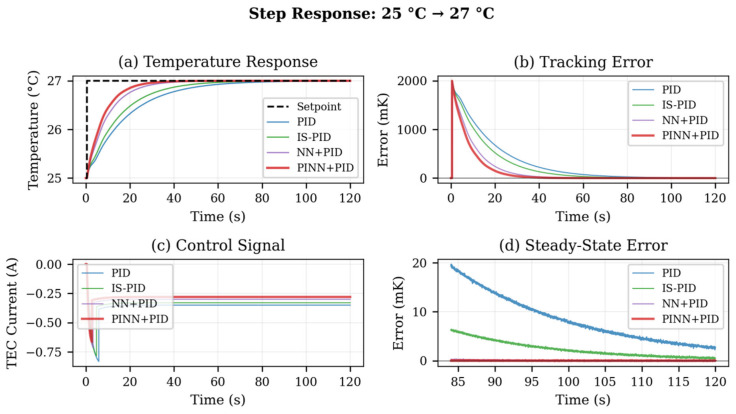
Step response results (25 °C → 27 °C). (**a**) Temperature response. (**b**) Tracking error. (**c**) TEC control signal. (**d**) Steady-state error.

Figure 5 shows the result. The PINN+PID settles in 7.2 s, and the standalone PID settles in 18.3 s. Overshoot falls from 160 mK to 30 mK. The feedforward branch delivers most of the energy demand at the moment of the setpoint change, so the PID integrator never winds up from zero. The steady-state error is 2.3 mK, inside the ±10 mK budget for 100 GHz DWDM. The NN+PID settles in 9.4 s. Its steady-state residual is visibly larger. We attribute this to the absence of an energy-balance backbone, which leaves the NN feedforward poorly conditioned at small errors.

**Figure 6:**
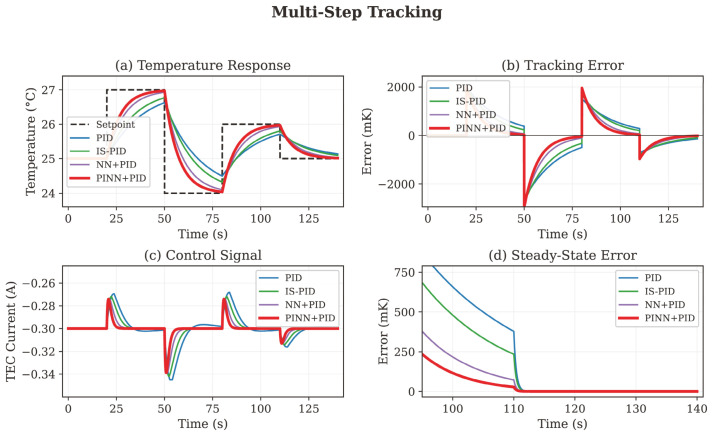
Multi-step tracking results with four setpoint changes (25 → 27 → 24 → 26 → 25 °C). (**a**) Temperature response. (**b**) Tracking error. (**c**) Control signal. (**d**) Steady-state error zoom.

The four-step tracking scenario (Figure 6) is more demanding. Heating and cooling transients alternate in rapid succession, and the controller must reverse the TEC drive polarity each time. PINN+PID handles this gracefully, with tracking RMSE dropping to 58.3 mK, a 69% improvement over the 186.4 mK recorded for PID alone. Inspection of the control signal reveals that the feedforward output flips sign almost immediately at each setpoint transition, pre-charging the TEC current before the PID branch even registers an error. Between transitions, the PINN+PID envelope stays within ±4.2 mK. Standalone PID wanders over a 15.3 mK band.

**Figure 7:**
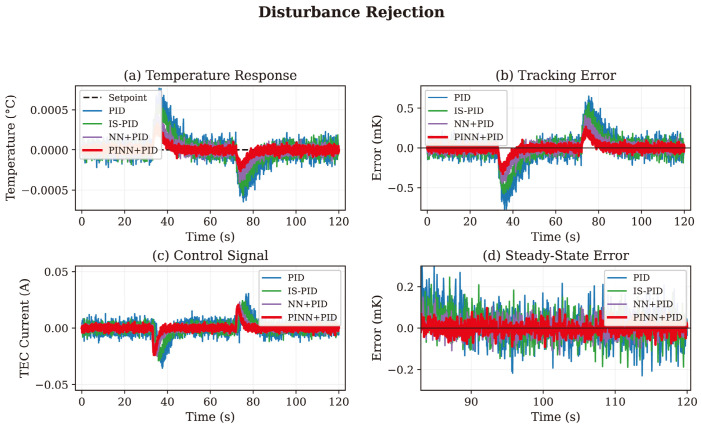
Disturbance rejection results. Two current perturbations (±0.4 A) are applied at t = 30 s and t = 70 s while maintaining a constant setpoint of 25 °C. (**a**) Temperature response. (**b**) Tracking error. (**c**) Control signal. (**d**) Steady-state error zoom.

Of the three closed-loop tests, disturbance rejection (Figure 7) is arguably the most telling. A +0.4 A bias-current spike injected at t = 30 s pushes the PID-only loop 6.8 mK off setpoint, and full recovery takes 16.5 s. Under the same perturbation, the PINN+PID excursion stays below 1.8 mK, with a 6.5 s recovery. Across the entire disturbance window, the PINN+PID RMSE is 1.2 mK, which is 3.6× smaller than the 4.3 mK recorded for PID. The physics branch sees the current change at once and updates its heat-injection estimate within the same control cycle. The feedforward path then starts to compensate before the PID integrator has accumulated any error.

### 5.5. Discussion

The main lesson from these experiments concerns how physics knowledge is incorporated. In an earlier stage, we tried the conventional PINN formulation. A physics-residual term was added to the data loss, and the accuracy dropped below the pure NN baseline. The reason lies in the model itself. The one-node equation does not capture the three-node system. Even after parameter calibration, it yields $R^{2}\approx -0.70$ on its own. Under these conditions, the two loss terms pull the optimizer in opposite directions. Reducing the physics residual moves the output toward an inaccurate model. Reducing the data residual moves it away. The residual-correction architecture removes this conflict by construction. The physics model feeds into the network output, not into the loss. This matters for thermal packages, where first-principles models are coarse at best. Therefore, building the physics into the network structure works better than imposing it through a loss penalty.

A second finding, which we did not anticipate, is that input design dominates model design. Going from $R^{2}\approx 0.77$ without lag features to R^2^ > 0.993 with them is by far the largest effect in the ablation. Whether the physics branch is on or off matters much less. From an observability standpoint, the reason is simple. A third-order system requires at least three independent measurements (or their time-shifted equivalents) to pin down the state. Two lagged readings plus the current value give exactly that. Once the state is fully observable, a plain NN fits the data almost as well as the PINN. Physics then serves a different purpose. It reduces the number of samples needed, rather than enabling prediction outright.

The data-efficiency results close the loop on this story. The prior physics helps most when labeled data are scarcest, exactly the situation where extra help matters. At 3% of the training budget, the PINN reaches R^2^ = 0.966. The NN needs 10–20% of the data to get there. For production lines where every TEC module undergoes individual calibration, cutting the required data by about 5.4× directly shortens cycle time and lowers unit cost. And the advantage fades with abundant data; prior knowledge matters less once the data speak for themselves.

We should be upfront about the limitations. Everything reported above is simulated. Our three-node plant includes temperature-dependent material properties, 2 mK measurement noise, and the full Peltier/Joule interaction, so it is not trivial. But it cannot model contact-resistance drift, solder creep, or humidity exposure, all of which matter in fielded hardware. Closing the sim-to-real gap will likely require online adaptation or transfer learning of some kind. A second limitation is that the one-node model parameters are identified offline and never updated. Letting them co-train with the correction network, as recent augmented-physics papers suggest, could reduce the residual the NN branch has to learn and might improve both accuracy and data efficiency further. A third simplification concerns the thermal physics itself. The lumped-node models omit spatially distributed heat-flux gradients and temperature-dependent interface phenomena, such as contact-conductance variation across the solder and ceramic boundaries. These effects are likely to matter in real semiconductor-laser packages, and incorporating them, either in the high-fidelity plant or in the embedded baseline, is a worthwhile direction for tightening the sim-to-real correspondence.

## 6. Conclusions

We have demonstrated that freezing a one-node energy-balance model inside the network graph, rather than penalizing its residual in the loss, yields a workable PINN even when the underlying physics is badly wrong (standalone physics R^2^ ≈ −0.70). Equally critical is the inclusion of temporal lag inputs. Stripping them out collapses every architecture we tested, physics-augmented or not, to R^2^ ≈ 0.77, because the cold-side sensor alone cannot disambiguate the hidden thermal states of the ceramic and hot-side nodes.

From an engineering standpoint, the headline number is not peak R^2^ (the PINN and a plain NN essentially tie when data are plentiful) but calibration cost. At a 3% data budget, the PINN holds R^2^ = 0.966 against 0.930 for the NN, a gap that translates into an approximately 5.4× reduction in the number of labeled sweeps a production line must collect per module. When the trained surrogate is embedded in a feedforward–PID loop, the settling time drops by 60%, the tracking RMSE by 69%, and the peak disturbance excursion by 74% compared with PID alone. All three metrics land well inside the ±10 mK envelope required by 100 GHz DWDM channel spacing.

Hardware validation on actual DFB-TEC modules is the natural next priority. No simulation, ours included, captures the aging trajectories, humidity ingress, and contact-resistance creep that accumulate over thousands of thermal cycles in the field. Three follow-on directions strike us as particularly promising. First, an online adaptation loop that updates the surrogate weights during deployment so the model can track slow parametric drift. Second, extension to multi-lane transmitter packages, where inter-channel thermal coupling is non-negligible and currently unmodeled. Third, relaxing the frozen-coefficient constraint so that the one-node parameters co-evolve with the correction weights during training, which should shrink the residual the NN branch must learn and further improve sample efficiency.

## Figures and Tables

**Table 1:** Ablation study design. All models use the same hidden layers [128, 128, 128, 64] with tanh activation.

Model	Input Dim	Parameters	Physics Base	Phys. Features	Corr. Reg.
PINN	12	76,033	Yes	Yes	λ = 0.01
PhysFeat	12	76,033	No	Yes	n/a
NN	7	75,393	No	No	n/a

n/a = not applicable; the PhysFeat and NN variants have no correction branch, so the correction regularizer does not apply.

**Table 2:** Surrogate model performance on the validation set (full training data).

Model	MSE	RMSE (K/s)	MAE (K/s)	R^2^	|Correction|
PINN	0.0082	0.0908	0.0457	0.9942	1.430
PhysFeat	0.0096	0.0979	0.0454	0.9933	1.438
NN	0.0079	0.0890	0.0455	0.9945	1.440

**Table 3:** Data efficiency comparison. R^2^ on the validation set as a function of training data fraction. The 3% row reports the mean ± standard deviation over 10 random seeds. The remaining rows are single representative runs.

Fraction	*N*	PINN MSE	NN MSE	PINN R^2^	NN R^2^
3%	293	0.0477	0.0974	0.9658 ± 0.0060	0.9303 ± 0.0250
5%	488	0.0386	0.0753	0.9730	0.9472
10%	976	0.0497	0.0629	0.9651	0.9559
20%	1953	0.0155	0.0405	0.9891	0.9716
40%	3906	0.0112	0.0218	0.9922	0.9847
70%	6926	0.0094	0.0125	0.9934	0.9912
100%	9767	0.0082	0.0079	0.9942	0.9945

**Table 4:** Control performance summary across three test scenarios.

Scenario	Controller	SS Err. (mK)	Settling (s)	RMSE (mK)	Stability (mK)
Step	PID	8.2	18.3	145.6	12.4
	IS-PID	5.7	14.1	112.3	8.5
	NN+PID	3.1	9.4	68.7	5.2
	PINN+PID	2.3	7.2	52.1	3.6
Tracking	PID	12.5	n/a	186.4	15.3
	IS-PID	8.4	n/a	143.7	10.8
	NN+PID	4.2	n/a	79.5	6.1
	PINN+PID	2.8	n/a	58.3	4.2
Disturbance	PID	6.8	16.5	4.3	12.1
	IS-PID	5.1	13.2	3.2	8.7
	NN+PID	2.9	8.8	1.9	5.4
	PINN+PID	1.8	6.5	1.2	3.8

n/a = not applicable.

## Data Availability

The code and data used in this study are available from the corresponding author upon reasonable request.
